# Anti-oxidant effect of gold nanoparticles restrains hyperglycemic conditions in diabetic mice

**DOI:** 10.1186/1477-3155-8-16

**Published:** 2010-07-14

**Authors:** Selvaraj BarathManiKanth, Kalimuthu Kalishwaralal, Muthuirulappan Sriram, SureshBabu Ram Kumar Pandian, Hyung-seop Youn, SooHyun Eom, Sangiliyandi Gurunathan

**Affiliations:** 1 Department of Biotechnology, Division of Molecular and Cellular Biology, Kalasalingam University, Anand Nagar, Krishnankoil-626190, Tamilnadu, India; 2 School of Life Sciences, Systems Biology Research Center, Gwangju Institute of Science and Technology, Gwangju 500-712, South Korea

## Abstract

**Background:**

Oxidative stress is imperative for its morbidity towards diabetic complications, where abnormal metabolic milieu as a result of hyperglycemia, leads to the onset of several complications. A biological antioxidant capable of inhibiting oxidative stress mediated diabetic progressions; during hyperglycemia is still the need of the era. The current study was performed to study the effect of biologically synthesized gold nanoparticles (AuNPs) to control the hyperglycemic conditions in streptozotocin induced diabetic mice.

**Results:**

The profound control of AuNPs over the anti oxidant enzymes such as GSH, SOD, Catalase and GPx in diabetic mice to normal, by inhibition of lipid peroxidation and ROS generation during hyperglycemia evidence their anti-oxidant effect during hyperglycemia. The AuNPs exhibited an insistent control over the blood glucose level, lipids and serum biochemical profiles in diabetic mice near to the control mice provokes their effective role in controlling and increasing the organ functions for better utilization of blood glucose. Histopathological and hematological studies revealed the non-toxic and protective effect of the gold nanoparticles over the vital organs when administered at dosage of 2.5 mg/kilogram.body.weight/day. ICP-MS analysis revealed the biodistribution of gold nanoparticles in the vital organs showing accumulation of AuNPs in the spleen comparatively greater than other organs.

**Conclusion:**

The results obtained disclose the effectual role of AuNPs as an anti-oxidative agent, by inhibiting the formation of ROS, scavenging free radicals; thus increasing the anti-oxidant defense enzymes and creating a sustained control over hyperglycemic conditions which consequently evoke the potential of AuNPs as an economic therapeutic remedy in diabetic treatments and its complications.

## Background

Diabetes mellitus a lifelong progressive disease is a chronic metabolic disorder due to the relative deficiency of insulin secretion and varying degrees of insulin resistance and is characterized by high circulating glucose [1]. This disease has reached epidemic proportion among the challenging unresolved health problems of the 21st century. Around 230 million people worldwide have been affected by diabetes and around 366 million people are expected to get affected by 2030 [2]. Several pathogenic pathways are activated in diabetes among which reactive oxygen species (ROS) generated by high glucose levels is responsible for metabolic abnormalities and chronic complications [3]. A counteractive defense system that eliminates the ROS produced during normal oxidative metabolism is being maintained and any imbalance in the production and scavenging of ROS leads to excessive levels of either molecular oxygen or ROS, thus resulting in increased 'oxidative stress' [4]. Since numerous studies have demonstrated that oxidative stress, mediated mainly by hyperglycemia-induced generation of free radicals, contributes to the development and progression of diabetes and its complications, it will be an effective strategy to use antioxidants to ameliorate treatments for oxidative stress. The management of diabetic conditions by insulin therapy has several drawbacks like insulin resistance and in chronic treatment causes anaeroxia nervosa, brain atrophy and fatty liver. Thus an effective and economic therapeutic molecule capable of up drifting the treatments for diabetes mellitus, by controlling the oxidative stress induced by hyperglycemia, disquieting various metabolic pathways and thereby preventing the onset of complications is still the need of the era.

Discovery of new molecules and manipulating those available naturally in nanosize could be appealing for their greater potential to improve health care [5]. Several pharmacological companies have won approval from the Food and Drug Administration (FDA) for the use and development of nanotechnology-based drugs in the last few years.

Gold compounds have received great attention as an anti-inflammatory agents through their ability to inhibit expression of NF-kappa B and subsequent inflammatory reactions [6-8]. The immunomodulatory, antioxidative and restorative activity of Swarna Bhasma in cerebral ischaemic rats has revealed their perceptive application in the treatment of ischaemia and cerebral damages [9]. The major drawback of ionic gold lies on the fact that they are easily inactivated by complexation and precipitation thus limiting their desired functions in human system. Here zerovalent gold nanoparticles can be a valuable alternative replacing the potential of metallic gold [10]. Gold nanoparticles (AuNPs), an emerging nanomedicine is renowned for its promising therapeutic possibilities, due to its significant properties such as biocompatibility, high surface reactivity, resistance to oxidation and plasmon resonance[11]. The inhibitory activity of gold nanoparticles against VPF/VEGF165 induced proliferation of endothelial cells provides clear evidence over their therapeutic potential in the treatment of diseases like chronic infiammation, pathological neo-vascularization, rheumatoid arthritis, and neoplastic disorders [12]. The role of gold nanoparticles invading the treatment for various inflammatory diseases and other relative disorders that are context dependent, in orientation with the evidences towards the anti-oxidative effect of traditional gold in treatment of diseases, have affirmed the urge for the need of study over restorative effect of gold nanoparticles at conditions of, hyperglycemia leading to, oxidative stress which has not been revealed yet.

Hence the effect of biologically synthesized gold nanoparticles on streptozotocin induced diabetic mice at hyperglycemic conditions leading to oxidative stress, have been investigated in this study.

## Results

### Characterization of Au-NPs

**Figure 1:**
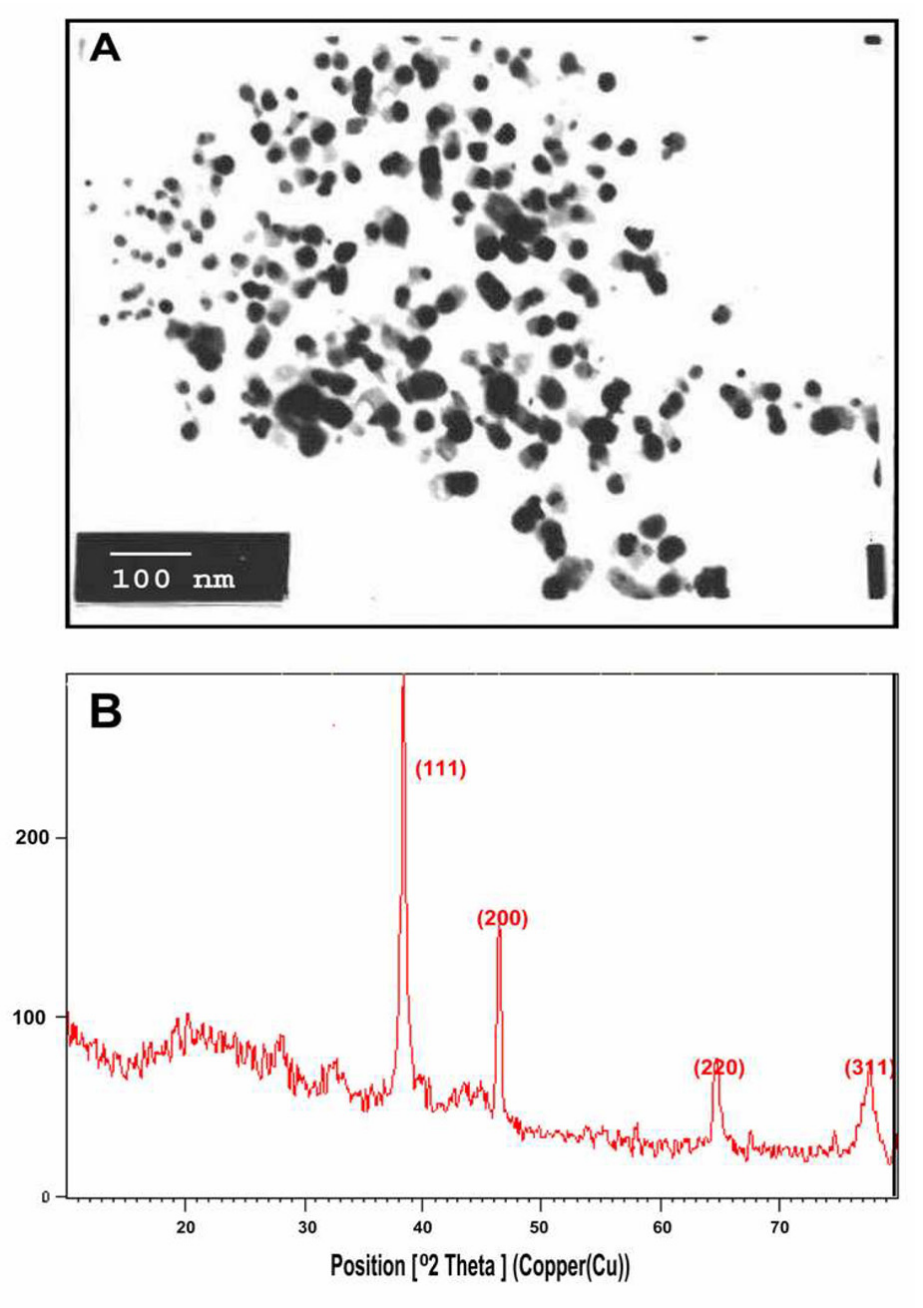
**A. TEM micrograph of the 1 mM AuCl_4 _^- ^ions-treated sonicated sample of *B. licheniformis *showing synthesized AuNPs**. Purified nanoparticles from *B. licheniformis *were examined by electron microscopy. Several fields were photographed and were used to determine the diameter of nanoparticles. The range of observed diameter of the synthesized gold nanoparticles was about 50 nm. **B. Representative XRD pattern of gold nanoparticles synthesized after θ24 h**. The XRD pattern shows four intense peaks in the whole spectrum of 2 θ values ranging from 20 to 80. Note 2 θ peak values of 39.01°, 46.48°, 64.69° and 77.62°, corresponding to 111, 200, 220, 311 planes, respectively, for gold.

Characterization of the synthesized gold nanoparticles was carried out before testing for their potent anti-oxidative effect in hyperglycemic conditions. The morphology and size of the biologically synthesized gold nanoparticles was determined using Transmission electron microscopy (TEM). The images clearly show that the average size of the particles was found to be in the order of 50 nm and depicts that they are relatively uniform in diameter and spherical in shape. (Figure 1A) The XRD pattern obtained showed four intense peaks in the whole spectrum of 2θ values ranging from 20 to 80. The presence of intense peaks of nanoparticles (111), (200), (220) and (311) appeared which are indexed as crystalline gold face centered cubic phase. The standard XRD patterns for Au are found to be almost similar [Joint Committee on Powder Diffraction Standards (JCPDS) file no: 01-1174 for Au]. The XRD pattern thus clearly shows that the gold nanoparticles formed by the reduction of AuCl_4 _^- ^ions by *Bacillus licheniformis *are crystalline in nature (Figure 1B). The Lal test revealed that the synthesized nanoparticles were endotoxin free based on the qualitative analysis which did not show any formation of gel clot.

### Toxicity studies

**Figure 2:**
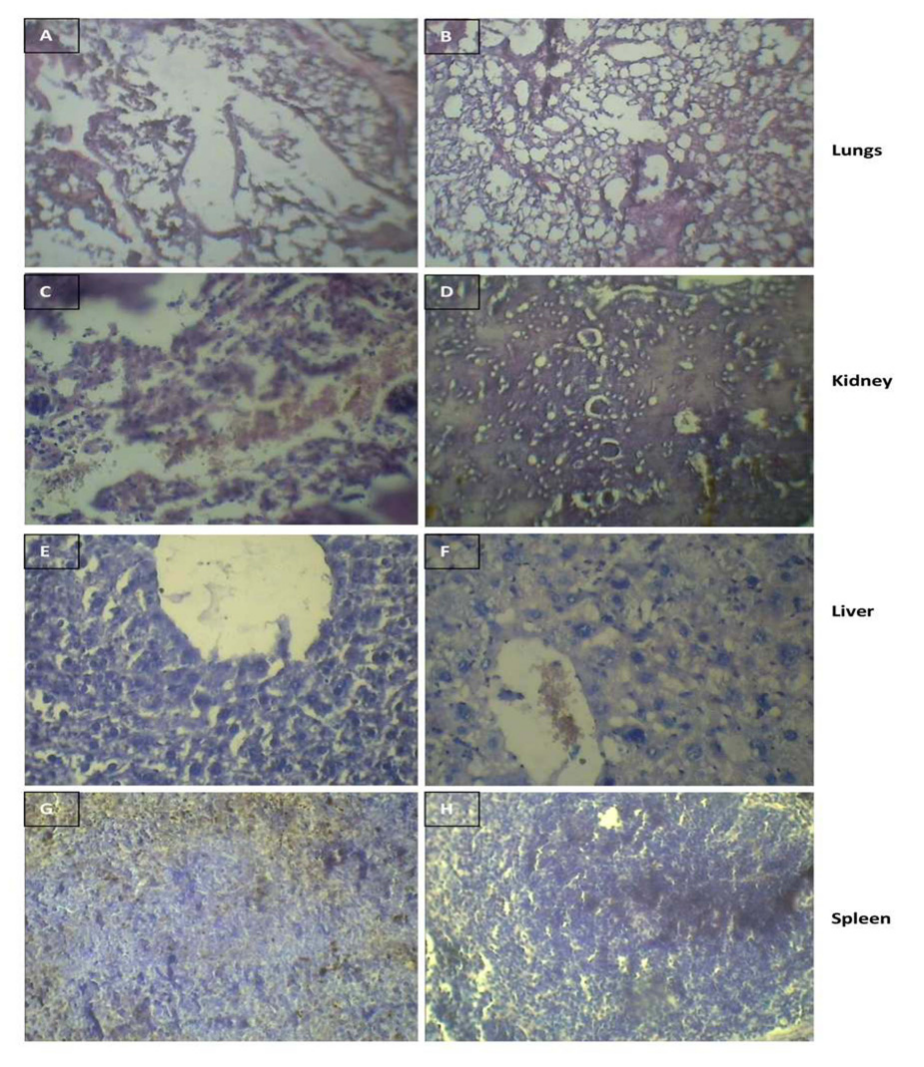
**Toxicity studies of gold nanoparticles in mouse organs**. Histological specimens of mice tissues (lung, kidney, liver and spleen) collected from mice euthanized on day 15, stained with hematoxylin and eosin (H and E) showed normal histology. 100% long-term survival of mice was also observed in the mice treated with gold nanoparticles at a concn of 500 nm for 15 days. A. Control animal lung section showing normal alveolar geometry and normal appearing alveolar septum. B. gold treated animal lung section showing normal alveolar membranes with normal parenchyma blood vessels. C. Control kidney section showing normal renal cortex and glomerular tufts D. gold treated kidney section showing normal glomerular tubules and renal cortex E. Control animal liver section showing normal, central vein and hepatocytic architecture F. Gold nanoparticles treated liver also showed normal hepatocytes with clear central vein G. Spleen sections of control animals showing normal splenic architecture with normal lymphoid follicles and sinuses H. gold treated spleen showing no pathological changes.

*In vivo *nanoparticles toxicity studies are focused mainly on examining changes in blood serum chemistry and cell population; changes in tissue morphology through histological analysis, along with nanoparticles biodistribution. These *in vivo *studies not only provide the toxicity information unavailable through *in vitro *studies but also inform the choice of relevant model system for carrying out further *in vitro *studies [13]. Thus the mice were injected with AuNPs at a dosage of 2.5 mg/kg.b.wt/day for 15 days and daily examined for any changes in the morphology and behavior. All the mice survived throughout the experimental period without exhibiting any abnormalities. The mice did not show any symptoms of toxicity such as fatigue, loss of appetite, change in fur color, weight loss, etc. Comparative analysis of various hematological parameters in the gold treated and control animals, clearly showed that there was no significant alteration except marginal variations in certain parameters (Table 1). Histological studies are well thought-out to be a reliable method to detect morphological changes due to toxicities. These histological/histopathological assays provide evidences over the morphological changes, evidencing that toxicity correlates with changes in tissue and cell morphology of a scale that can be visualized using light microscopy [14]. Thus the pathological effect of the nanoparticles over the morphological characteristics of the organs was examined through the histological observations using light microscope. The histopathological findings of the non-toxic effect of the gold nanoparticles over liver, kidney, spleen and lung that were observed are presented in (Figure 2). The examined reports obtained from the senior pathologist confirmed that the gold nanoparticles treated organs did not show any significant morphological changes in comparison to control. In the lung histopathology the sections from control animals was showing normal alveolar geometry and normal appearing alveolar septum (Figure 2A). The same histopathological finding was seen after the treatment of gold nanoparticles at a concn of 500 nm day^-1 ^(Figure 2B) showing normal alveolar membranes with normal parenchyma blood vessels. The kidney histological studies showed the control kidney with normal renal cortex and glomerular tufts (Figure 2C) and the treatment of gold nanoparticles at a dosage of 2.5 mg/kg.b.wt/day did not lead to any disruptions in the histology. They showed normal glomerular tubules and renal cortex (Figure 2D). In the Liver histopathology sections from the control animals are showing normal hepatic portal triad and central vein (Figure 2E). The gold nanoparticles treated liver also showed normal hepatocytes with clear central vein showing no morphological changes significant in comparison to control (Figure 2F). The study over the spleen histology also revealed that there were no any disruptions due to the treatment of gold nanoparticles at a dosage of 2.5 mg/kg.b.wt/day. The control and gold treated spleens showed normal lymphoid follicles and sinuses (Figure 2G-H).

**Table 1:** Hematological analysis revealing the nontoxic effect of AuNPs in mice

Parameters	Control	Gold treated
Hb (g/dl)	10.8 (± 1.19)	11.10 (± 1.3)
RBC Distrib Width (%)	17.4 (± 3.1)	17.95 (± 2.5)
MCV (fL)	48.30 (± 0.8)	44.0 (± 0.66)
MCH (pg)	24.75(± 5.33)	26.3(± 4.89)
MCHC (g/dl)	32.10(± 0.43)	33.8(± 0.28)
Platelet count [× 10^(9)^/L]	289(± 39.72)	298(± 44.69)
RBC [× 10^(12)^/L]	4.22((± 0.15)	4.74((± 0.3)
Leukocytes[× 10^(9)^/L]	2.74(± 0.6)	3.91(± 0.8)
HCT (%)	31.32((± 2.4)	33.0((± 1.55)

Each value represents the mean ± S.D of n = 6 Hb, hemoglobin; cells; RBC, red blood cells; MCV, mean corpuscular volume; MCH, mean corpuscular hemoglobin; MCHC, mean corpuscular hemoglobin content; HCT, hematocrit. Numerical values (±) in the parenthesis are considered as 'Standard Deviation (SD)'. P values were calculated using one way ANOVA followed by Students-'t' test by comparing between different groups (control vs. treatment) and values are considered to be non-significant (P > 0.05).

### Blood parameters

**Figure 3:**
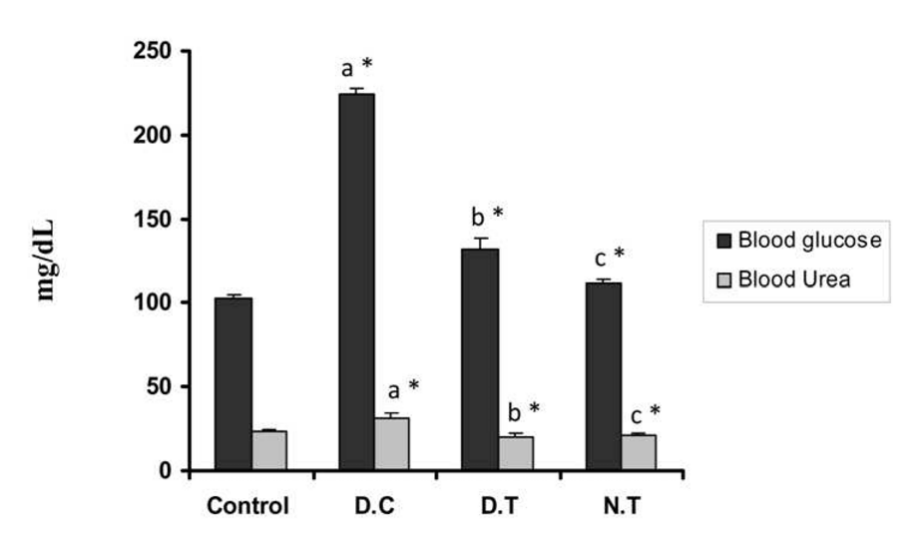
**Control effect of gold nanoparticles over blood glucose and urea in experimental mice**. The treatment of gold nanoparticles significantly restrained the blood glucose and urea level to normal near to control in comparison to diabetic control. Datas are given as mean ± S.D for n = 6. Values are statistically significant at * p < 0.05.^a ^Diabetic control compared with control group.^b^Gold treated diabetic group compared with diabetic control group.^c^Gold treated group compared with control group.

The control effect of gold nanoparticles over the blood glucose and blood urea level obtained is represented in Figure 3. The blood glucose level increased two fold and blood urea level were observed to be elevated significantly in the diabetic control mice in comparison to control group. The diabetic treated group showed a controlled effect over the induced hyperglycemic condition by significantly decreasing the blood glucose by 75% in comparison to the diabetic control. The blood glucose and Urea level of gold treated group also did not show any significant changes in comparison to the control group (p < 0.05).

Various parameters of blood lipid profile were tested in streptozotocin-induced diabetic mice before and after the treatment with the gold nanoparticles. Treatment with gold nanoparticles lowered the levels of TC, LDL, VLDL and TG in diabetic mice near to normal. The level of TC, LDL cholesterol and TG, were significantly decreased at about 55%, 65% and 45% respectively in diabetic mice treated with gold nanoparticles as compared to diabetic control. Similarly, HDL levels were found to be increased partially in diabetic mice after the treatment with gold nanoparticles as compared to diabetic control. (p < 0.05) (Table 2). The gold nanoparticles treated mice group IV did not show any significant changes in comparison to control group I.

**Table 2:** Control Effect of gold nanoparticles over the Lipid profile

Group	TC	HDLC	LDLC	TG	VLDLC
**Control**	93 ± 5.8	20 ± 2.0	55 ± 4.7	82 ± 3.6	16 ± 2.0
**Diabetic control**	84 ± 10^a^*	15.4 ± 1.9^a^*	134 ± 6.4^a^*	121 ± 9.6^a^*	22.4 ± 3.1^a^*
**Diabetic treated**	98 ± 4.3^b^*	25 ± 3.2^b^*	41.8 ± 5.1^b^*	82.6 ± 3.5^b^*	16.9 ± 1.7^b^*
**Gold treated**	99 ± 8.2^c^*	28 ± 1.7^c^*	56.8 ± 2.7^c^*	89.6 ± 6.0^c^*	17.9 ± 1.3^c^*

Datas are given as mean ± S.D for n = 6. Values are statistically significant at * p < 0.05.^a ^Diabetic control compared with control group.^b^Gold treated diabetic group compared with diabetic control group.^c ^Gold treated group compared with control group.

### OGTT

**Figure 4:**
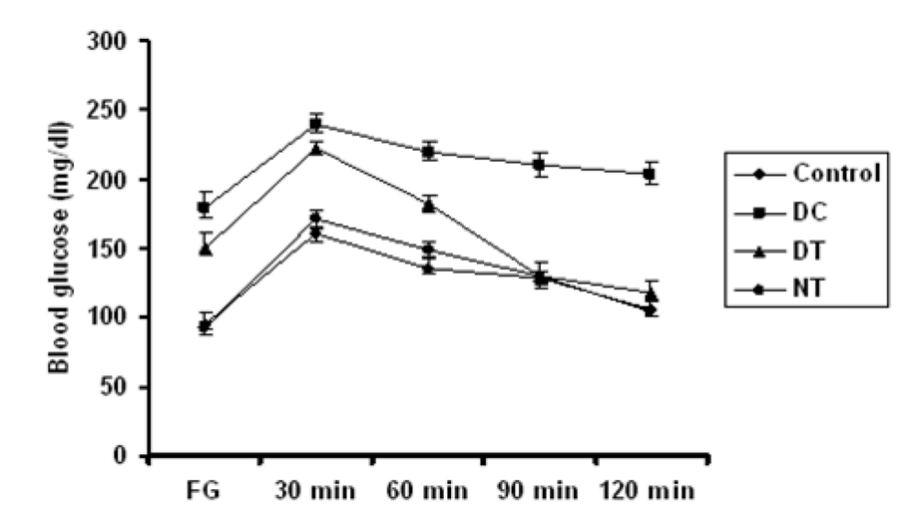
**Oral glucose tolerance test (OGTT)**. The glucose tolerance of streptozotocin-induced diabetic mice in response to gold nanoparticles treatment. The ability of gold nanoparticles to maintain the blood glucose of the diabetic treated mice near to the control in various time intervals is shown. Results are means ± S.D of n = 6. FBG, fasting blood glucose.

The control effect of the gold nanoparticles over high glucose conditions was studied by Oral Glucose Tolerance Test (OGTT). The blood glucose level at fasting conditions (FBG) and after the oral administration of glucose in control and experimental animals are represented in (Figure 4). Blood glucose levels, estimated in overnight fasting diabetic mice (FBG), were significantly elevated. However, this level was reduced significantly upon treatment with gold nanoparticles at a dosage of 2.5 mg/kg.b.wt/day (Figure.4, FBG data). For GTT, 1 g/kg.b.wt of glucose dissolved in water were fed to the overnight-fasted mice and the blood glucose level was determined up to 120 min. The blood glucose level had decreased significantly by 90 min in comparison with the elevation by 30 min and this was maintained until 120 min with an effective dose of gold nanoparticles (p < 0.05).

### Serum analysis

The enzymes such as ALT, AST, ACP and ALP are responsible for the proper functioning of the liver and any damages induced in the liver due to the hyperglycemic conditions may lead to excessive leakage of these enzymes in the blood stream. Thus the effect of gold nanoparticles over the level of different metabolic enzymes shaping the effective functioning of the liver through the serum analysis was analyzed and their protective effect of gold nanoparticles over the liver damage is shown in Table 3. The enzymes ALT, AST, ACP and ALP showed significant elevated levels in the diabetic control group (G2) in comparison to control group. Following treatment of gold nanoparticles at a dosage of 2.5 mg/kg. b. wt, the diabetic treated group (G3) presented a partial decrease significantly in comparison to the diabetic control group (G2), which directly reveals the protective/regenerative effect over the exaggerated activity of liver. The level of creatinine symptomatic of the renal functions was also decreased significantly near to normal in the diabetic treated groups in comparison to the diabetic control group. The gold nanoparticles treated mice did not show any significant changes of creatinine level in comparison to the control (p < 0.05). These results obtained over the restorative effect of gold nanoparticles over the metabolic enzymes confirm the ability of gold nanoparticles to protect the organs from damage due to hyperglycemia induced oxidative stress.

**Table 3:** Effect of gold nanoparticles over the metabolic enzymes

Group	AST	ALT	ALP	ACP	creatinine
**Control**	14.3 ± 0.64	12.17 ± 1.11	43.6 ± 1.78	5.18 ± 0.13	0.1 ± 0.02
**Diabetic control**	34.72 ± 1.12^a^*	22.67 ± 2.96^a^*	76.92 ± 2.06^a^*	9.76 ± 0.37^a^*	3.82 ± 0.24^a^*
**Diabetic treated**	15.8 ± 0.89^b^*	13.4 ± 1.34^b^*	53.4 ± 0.71^b^*	7.32 ± 0.19^b^*	0.49 ± 0.01^b^*
**Gold treated**	18.91 ± 5.01^c^*	15.13 ± 0.62^c^*	46.02 ± 0.61^c^*	5.92 ± 0.14^c^*	0.77 ± 0.15^c^*

Results are given as mean ± S.D (n = 6). Values are statistically significant at *p < 0.05.^a ^Diabetic control compared with control group.^b ^Gold treated diabetic group compared with diabetic control group.^c ^Gold treated group compared with control group. All the values of AST, ALT, ALP and ACP are expressed as IU/L and creatinine in mg/dL.

### ROS generation and lipid peroxidation

**Figure 5:**
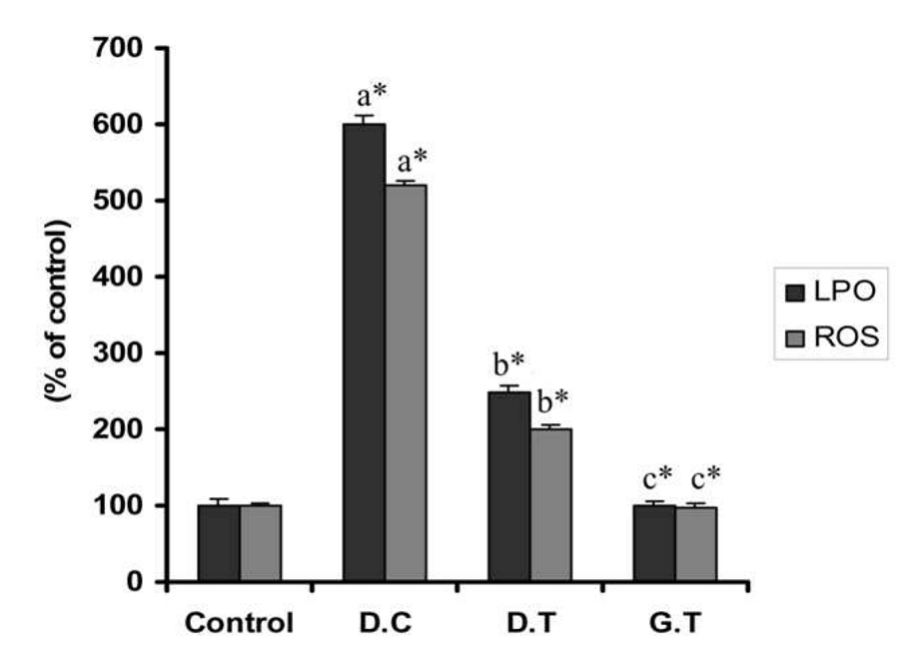
**Influence of gold nanoparticles over the anti-oxidant system in experimental mice**. The gold nanoparticles restored the elevated level of antioxidant enzymes such as GSH, SOD, GPx and catalase to normal. Values are expressed as mean ± S.D (n = 6). Values are statistically significant at *p < 0.05.

ROS generated by high glucose levels play a vital role in the development of diabetic complications [15]. It is the resultant of the oxidative stress developed due to the release of free radicals, thereby decreasing the level of antioxidant enzymes. Estimation of ROS generation in the liver revealed that gold nanoparticles blocked the high glucose-induced increase in ROS generation to a maximum extent in the liver which is shown in Figure 5. Induction of diabetes in the group II mice results in a twofold level of increase in ROS generation relative to the control mice. The diabetic mice treated with gold nanoparticles significantly decreased the high glucose-induced rise in ROS generation in the liver in comparison to diabetic control mice. This makes clear the inhibitory effect of gold nanoparticles over ROS generation during hyperglycemia induced oxidative stress.

Functional damage to cells under oxidative stress is not only by oxygen free radicals and unbalanced redox potential but also due to enhanced lipid peroxidation [16]. The inhibitory effect of gold nanoparticles over the occurrence of lipid peroxidation in the enzyme source is confirmed which is shown in Figure 5. A potent control effect of gold nanoparticles (500 nM) treated to the diabetic treated group showed a significant decrease in lipid peroxidation compared with diabetic control group mice. The gold nanoparticles treated normal mice did not show any significant elevation of the peroxidation in comparison to control (p < 0.05).

### Effect of gold nanoparticles over the Antioxidant system

Glutathione (GSH) is a tripeptide with a free reductive thiol functional group, responsible for the detoxification of peroxides such as hydrogen peroxide or lipid peroxides, and acting as an important anti-oxidant in cells. During the detoxification process GSH (reduced form) becomes oxidized glutathione (GSSG) which is then recycled to GSH by the enzyme glutathione reductase present in cells. The increased ROS levels in diabetes could be due to their increased production and/or decreased destruction by antioxidants such as GSH, SOD, catalase and glutathione peroxidase [17-21].

**Figure 6:**
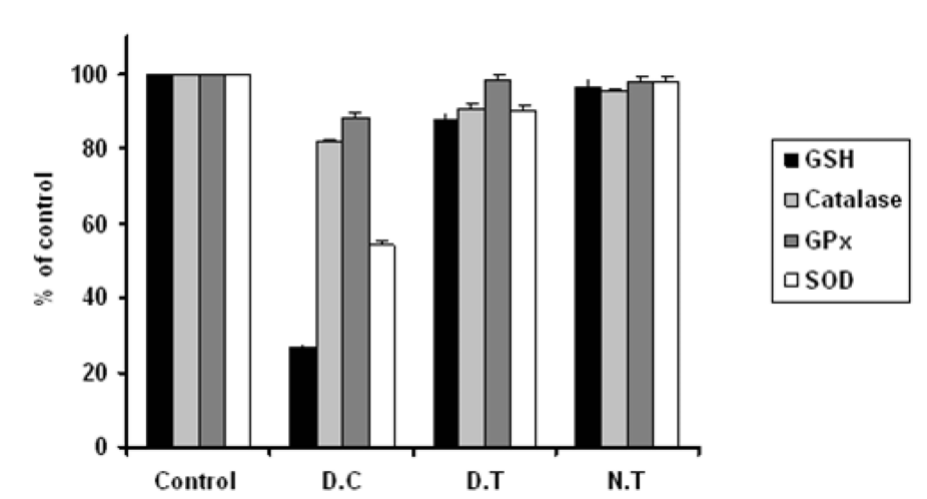
**Effect of gold nanoparticles over the ROS generation and Lipid peroxidation in experimental mice**. The gold nanoparticles inhibited increased ROS generation and Lipid peroxidation thereby restoring the anti-oxidant system to normal. Datas are given as mean ± S.D for n = 6. Values are statistically significant at * p < 0.05. ^a ^Diabetic control compared with control group. ^b ^Gold treated diabetic group compared with diabetic control group. ^c ^Gold treated group compared with control group.

To define the molecular mechanisms of the anti-oxidative effect of gold nanoparticles due to high glucose-induced oxidative stress in the mice, the effects of gold nanoparticles on GSH levels in the diabetic treated mice were investigated. GSH levels were measured, and shown in Figure 6 stating that GSH levels increased significantly in the diabetic control group relative to the control group mice treated with citrate buffer alone. The GSH levels reached a plateau when treated with AuNPs at dosage of 2.5 mg/kg.b.wt/day in comparison with diabetic control. These results suggest that gold nanoparticles could exert cytoprotective effects on diabetic mice through the stimulation of GSH activity.

SOD is responsible for the catalysis of the dismutation of the superoxide anion into hydrogen peroxide and molecular oxygen. The cellular levels of SOD were significantly turned down in the diabetic group mice as compared with the control group. Compared with the diabetic control group, diabetic treated group, treated with AuNPs showed the significant increase in the SOD activity to 80% that was near to normal (p < 0.05) (Figure 6).

The catalase and Glutathione peroxidase that are considered as primary anti-oxidants responsible for the direct elimination of ROS generated. A significant decline in the level of the enzymes respectively in the diabetic group mice as shown in Figure 6, were restored near to control through a significant increase in the diabetic treated mice with gold nanoparticles (p < 0.05).

### Histopathological studies

**Figure 7:**
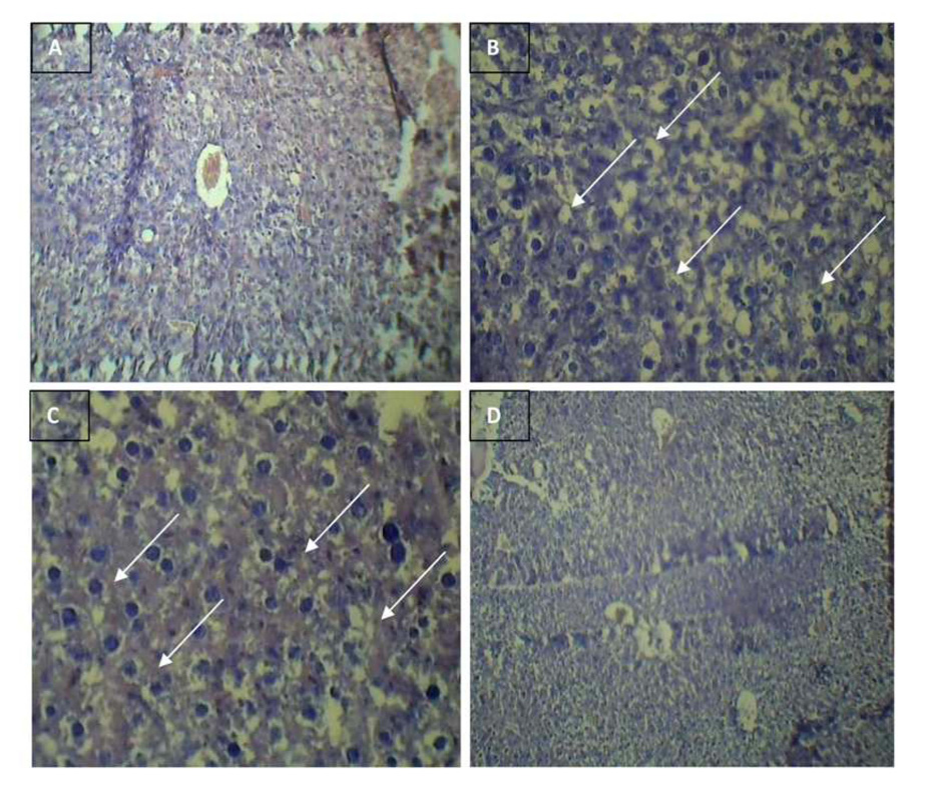
**Protective effect of gold nanoparticles over hyperglycemia induced liver damage in diabetic mice**. Histological specimens of mice liver after treatment of gold nanoparticles for 45 days in experimental group of mice revealing the preventive effect of gold nanoparticles over oxidative stress induced organ damage in the liver. A. control liver showing normal hepatic architecture, portal traid and central vein B. diabetic control liver showing ground glass nuclei, lymphocytic infiltrations along with lobular inflammation and high fatty cells C. diabetic treated liver showing a significant reduction in the fatty cells near to normal along with a clear central vein. D. gold treated liver for 45 days showing whole nuclei with central vein without any significant morphological disruptions.

**Figure 8:**
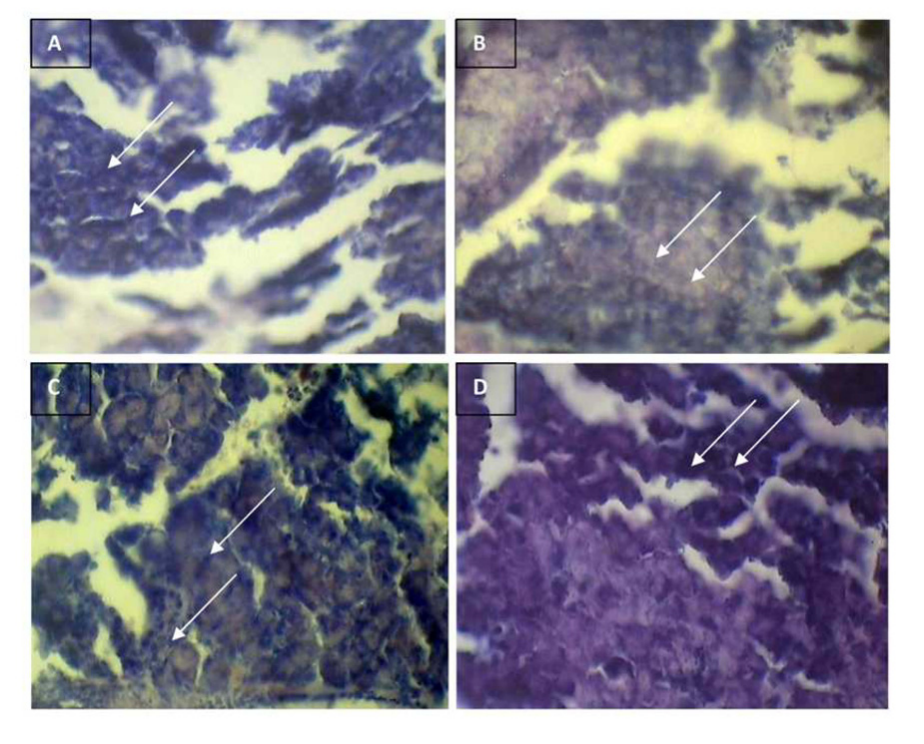
**Protective effect of gold nanoparticles over hyperglycemia induced damage in pancreas of diabetic mice**. Histological sections of pancreas of experimental group of mice after treatment with gold nanoparticles for 45 days revealing the preventive effect of gold nanoparticles over oxidative stress induced organ damage in the pancreas. A. normal islets with clusters of purple stained β-cells B. the greater atrophy of β-cells and vascular degeneration C. increased size of β-cells and clear islets near to normal D. normal atrophy of pancreatic cells similar to normal without any degenerative effects.

Histological analysis over the liver and pancreas was carried out in order to examine the potency of gold nanoparticles to prevent the organs from damage. The results obtained as shown in Figure 7 and 8, revealed the inhibitory and protective effect of gold nanoparticles over the organ damages at hyperglycemic conditions. The liver of the control mice showed normal hepatic architecture, portal traid and central vein (Figure 7A). The diabetic control mice showed ground glass nuclei and lymphocytic infiltrations along with lobular inflammation with high fatty cells (Figure 7B). The diabetic induced mice treated with gold nanoparticles showed a significant reduction in fatty cells, normal central vein with no ground glass nuclei with, stating the restorative effect of AuNPs over the organ damage. (Figure 7C). The gold nanoparticles treated mice also showed normal whole nuclei with central vein without any significant morphological disruptions in comparison to normal (Figure 7D). Sections of pancreas from the control group showed normal islets (Figure 8A). The diabetic control mice showed degeneration of pancreatic cells along with lymphocytic infiltration (Figure 8B) and the diabetic treated mice had clearly shown the protective effect of AuNPs with the clear area occupied by the β cells stating their regeneration (Figure 8C). The gold treated pancreas also did not exhibit any degenerative effects in the cells as shown in Figure 8D.

### Biodistribution of gold nanoparticles

**Figure 9:**
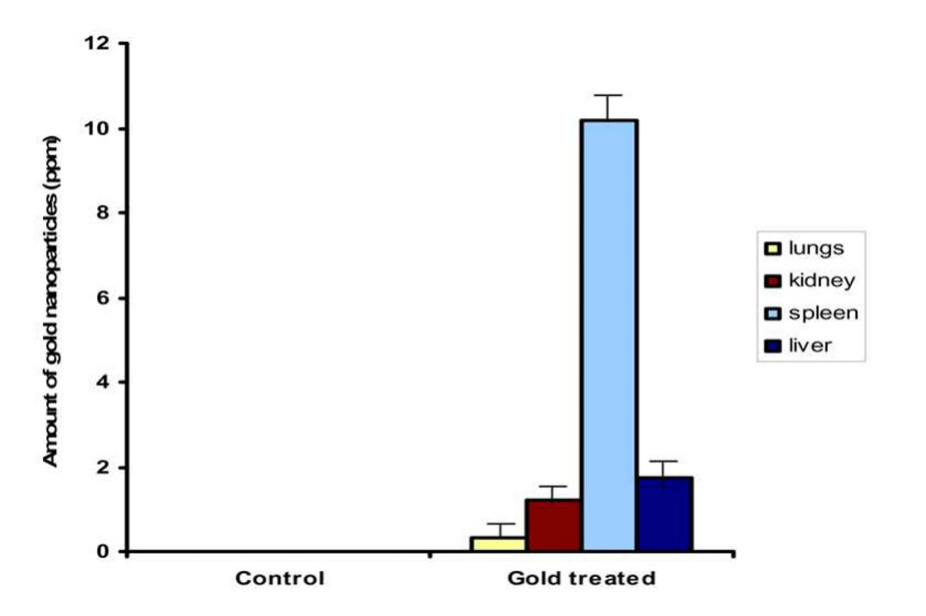
**Biodistribution of gold nanoparticles in mice**. ICP MS data shows the biodistribution of gold nanoparticles in different organs (lungs, kidney, spleen, liver) of mice euthanized (toxicity study) after treatment with gold nanoparticles suspended in deionized water for fifteen days through intra-peritoneal injection which reveals the greater accumulation of gold nanoparticles in the spleen comparatively higher than in other vital organs. Values are statistically significant at p < 0.05.

The distribution of gold element was detected in diverse organs such as liver, kidney, spleen and lungs using the ICP-MS. The gold nanoparticles were distributed in all organs, and the distribution pattern obtained is shown in Figure 9. The concentration of gold element in different organs was analyzed by inductively coupled plasma mass spectrometry (ICP-MS). The biodistribution of gold element (per gram of tissue) in different organs of control and gold treated mice after intra-peritoneal injection during the treatment are shown in Figure 9. The accumulated gold concentration in spleen, lungs, kidney and liver was found to be 10.19, 0.32, 1.21, 1.74 ppm of the tissue by volume respectively.

## Discussion

The promising potential of gold nanoparticles in treating inflammatory and auto immune diseases [22] have augmented greater interest to investigate the anti-oxidative and anti-hyperglycemic activity of the gold nanoparticles in the diabetic system.

In this study the gold nanoparticles were biologically synthesized by slight modification in the method described earlier [23]. In the previous method biological gold nanocubes are synthesized using nitrate media as a prime source at optimum alkaline pH whereas in the present study the use of nutrient media replacing the nitrate media, at working pH 7 is responsible for the synthesis of spherical gold nanoparticles. The results obtained in the synthesis and characterization of the synthesized nanoparticles is strongly supported by previously published reports on synthesis of silver nanoparticles using the same biological method and strain [24].

The preliminary objective of the study was to confirm the nontoxic nature of the biologically synthesized gold nanoparticles of size 50 nm in the *in vivo *system. Cells are capable of taking up gold nanoparticles without any cytotoxic effects [25] and in case PEG modified gold nanorods removing the stabilizer CTAB did not show any cytotoxicity [26]. The nontoxic effect of the gold nanoparticles in the present study was confirmed for no sub clinical toxicology through hematological analysis and histological studies over the vital organs (liver, kidney, spleen and lung) after the administration of gold nanoparticles for 15 days, which is supported by the evidence over the size dependent toxicity of gold nanoparticles in experimental mice that revealed the acute toxic effects of gold nanoparticles of size range about 8, 17, 12 and 37 nm over the mice, whereas gold nanoparticles of size ranging about 3, 5, 50 and 100 nm did not show signs of any toxic effects [27]. Our results corroborate with the previous researches made by Hainfeld et al [28] in using gold nanoparticles as advantageous X-Ray contrast agent than other existing chemical contrasts in which the gold nanoparticles exhibited a non-toxic effect over the blood chemistry and vital organs. Recently the anti-glycation activity of gold nanoparticles in addition to their biocompatibility has made them preferable for ophthalmological implications [29]. Therefore in the present study, after confirmation of the non toxic nature of the AuNPs of size 50 nm, the effect of the gold nanoparticles over the oxidative stress induced at hyperglycemic conditions was investigated, which auspiciously showed the significant reduction of peak levels of sugar within two hours during GTT that strengthens the anti-diabetogenic potential of the gold nanoparticles in the mice model. Further, the AuNPs at a dosage of 2.5 mg/kg.b.wt significantly decreased the blood glucose level and the blood urea level at a range compared to the diabetic control groups when analyzed for the blood parameters in consistent to the previous researches made.

Hypertriglyceridemia which is a widespread finding in patients with diabetes mellitus and plays a leading role in vascular complications [30]. The treatment of gold nanoparticles in the diabetic mice for a period of 45 days have restored the total cholesterol and the triglycerides levels near to the normal thus resuming lipid functioning similar to that of non diabetic control group. The enzymes ALT (SGPT), AST (SGOT), ALP and ACP are the metabolic enzymes which leak into the blood stream during liver damage due to oxidative stress and the potential of AuNPs to control these enzymes to normal affirm their ability to prevent the organs from damage. ALP is also called cholestatic liver enzymes. Chloestasis is a condition that causes partial or full blockage of the bile ducts [31]. Bile ducts bring bile from the liver into the gall bladder and the intestines. Bile the green fluid produced in liver cells helps the body to break down fat, process cholesterol and get rid of toxins. If the bile duct is inflamed or damaged, ALP can get backed up and spill out from the liver into the bloodstream. This restorative activity of gold nanoparticles to normalize the bile action confirms the ability of gold nanoparticles to bring the lipid profile in the diabetic mice to normal which is consistent with its potential activities against inflammatory responses [22]. The level of creatinine which shows the normal functioning of renal activities was also restored near to normal in the diabetic treated mice that state the role of AuNPs in preventing the kidney from damage. These restorative and nontoxic effects of gold nanoparticles over the serum clinical chemistry correlate with previous evidences of researches made using gold nanoparticles in enhancement of radiotherapies in mice in which the mice treated with gold nanoparticles for 11 and 28 days did not exhibit any significant changes in comparison to the control [32].

The activities of antioxidant defense enzymes in charge for scavenging free radicals and maintaining redox homeostasis such as SOD and GSH are diminished during hyperglycemia. Increased glucose flux both enhances oxidant production and impairs antioxidant defenses by multiple interacting pathways [33]. In the present study a statistically significant increase in the levels of GSH, SOD, catalase and GPx in the diabetic treated mice with AuNPs in comparison to diabetic control is being proved which is due to the significant decrease in lipid peroxidation and ROS generation that was accomplished in diabetic treated mice with AuNPs, relative to diabetic control suggesting that AuNPs prevents disruption of organs by protecting lipids from peroxidation by ROS under hyperglycemic conditions.

Oxidative stress plays a foremost role in etiology of several diabetic complications [34-36]. The ability of gold nanoparticles in inhibiting the lipid from peroxidation thereby preventing the ROS generation has restored the imbalances in the antioxidants and liver enzymes responsible for the cell dysfunction and destruction, leading to tissue injury in the diabetic control group at hyperglycemic conditions. Our result suggesting gold nanoparticles' potential as antioxidant is shored up with previous reports delivering the control effects of gold nanoparticles as an antioxidant [37] and potential of other rare earth metals like cerium oxide to scavenge free radicals ROIs in retinal neurons [38]. These results are also supported by the findings that suggest the non-cytotoxic effect of Au(0) nanoparticles, and the ability of gold nanoparticles to reduce the production of reactive oxygen and nitrite species, which do not elicit secretion of proinflammatory cytokines TNF-α and IL1-β, making them suitable candidates for nanomedicine[39]. The potential ability of AuNPs in this study to inhibit the oxidative stress mediated ROS generation is highly supported by existing evidences of various other nanoparticles such as Platinum nanoparticles that had an immense ability to inhibit the pulmonary inflammation led by oxidative stress as a result of cigarette smoking due to their antioxidant properties[40]. The melatonin-selenium (MT-Se) nanoparticles also relapsed the ROS generated and Lipid peroxidation based on which their antioxidant effect is confirmed [41]. The advantage of our biologically synthesized AuNPs over these nanoparticles is that biologically synthesized nanoparticles have a greater stability and do not agglomerate or aggregate.

Histological studies carried out over the liver and pancreas for the four groups i.e. control, diabetic control, diabetic control treated and gold treated exposed the capability of AuNPs in restoring the organs to normal histology in the diabetic treated mice in comparison to the morphological disruptions in the diabetic control mice. Thus the gold nanoparticles reinstate the organ damages in the diabetic system by their sustained control over the ROS generation and inhibition of lipid peroxidation. Recent studies demonstrate that the primary and key event responsible for the activation of several pathways involved in the pathogenesis of diabetic complications is possibly a single hyperglycemia-induced process of overproduction of super oxide by the mitochondrial electron-transport chain seems [42]. Thus these findings over the ability of AuNPs in the elimination of ROS induced at hyperglycemic conditions, thereby restoring the balanced level of anti-oxidant defense system affirms the therapeutic application of gold nanoparticles as a promising anti-oxidant.

ICP-MS study carried out over the bio-distribution of gold nanoparticles in the different organs enriched with the reticulo endothelial system (RES) such as the liver, spleen, lungs and non-RES organs such as kidney of the mice revealed that the distribution of gold in liver, kidney and lungs was almost negligible which is not leading to any adverse effects in the system. The concentration of gold is significantly higher in the spleen as compared to other organs during the treatment period. Our results show that the gold nanoparticles are rapidly and widely redistributed in the body except in the case of the spleen thereby suggesting that the localization of the gold nanoparticles in the liver, lungs, and spleen was not consistent with the RES system. Long term studies performed in naive animals revealed that the accumulation of gold in the liver gradually cleared out over time with approximately 35% of the total injected Au present in the organs [43]. The clearance may be either via the urine or feces. It is reported that hydrodynamic size [44] of nanoparticles (NPs) also affects NPs clearance from circulation [45-47]. Studies over the various size dependent accumulation of gold nanoparticles have been reported which states that small NPs (< 20 nm) are excreted renally, [48] while medium sized NPs (30-150 nm) have accumulated in the bone marrow, [49] heart, kidney, and stomach; [48] and large NPs (150-300 nm) have been found in the liver and spleen [45]. In the present study the distribution of gold nanoparticles of 50 nm have been found, that particles do not to get accumulated in the kidney, stating that though these size ranges provide general clearance mechanisms, other physical parameters, clinical significance, and long-term persistence of gold nanoparticles simultaneously affecting NPs movement play a significant role in their distribution.

The potential of gold nanoparticles to restore the blood glucose and urea levels along with the biochemical profiles at hyperglycemic conditions arises various possibilities over their mechanism through which they act. There is no any single pathway by which oxidative stress is increased by diabetes-induced hyperglycemia [50]. Formerly, oxidative stress in diabetes mellitus has been linked to improved production of superoxide anion by mitochondria [51] and through protein kinase C-dependent activation of membranous NADPH oxidase [52]. Hyperglycemia has also been implicated in the activation of several stress-activated signaling pathways that include nuclear factor-B (NF-B), NH_2_-terminal Jun kinases/stress activated protein kinases (JNK/SAPK), p38 mitogen-activated protein (MAP) kinase, and hexosamine. Datas now indicate that activation of these pathways is linked not only to the development of the late complications of diabetes, but also responsible to insulin resistance and β-cell dysfunction [53]. The fullerene nanoparticles were known to selectively enter cells damaged due to oxidative stress and potentially inhibited apoptosis by hindering the JNK pathway [54]. Thus the potential antioxidant property of gold nanoparticles in controlling the oxidative stress mediated reactive oxygen species generation and lipid peroxidation which is being proven in the present study may be due to inhibitory activity of gold nanoparticles over one of the pathways above, which is to be revealed yet.

Another hypothesis that lies on the antioxidant property of gold nanoparticles is their interaction with the thioredoxin. Thioredoxin, a highly conserved thiol reductase that act over an endogenous inhibitor, thioredoxin-interacting protein (Txnip), is responsible for the antioxidative mechanism through the regulation of cellular redox balance. Txnip is present in abundance during hyperglycemic conditions and thus interaction of higher inhibitor proteins lead to several adverse effects in the anti-oxidants levels. The Gold nanoparticles are known to possess greater binding affinity to the cysteine residues and thus it may be possible that the gold nanoparticles replace the inhibitor binding to thiol reductase during hyperglycemia. The binding reaction between Au surface and cysteine residue in the protein is highly stable [55].

Hence the anti-oxidative and anti-hyperglycemic effect of gold nanoparticles along with their protective effect over the organ damage during conditions of hyperglycemia induced oxidative stress may be attained through the inhibition of the stress signaling pathways or, due to the interaction of the AuNPs to the cystein-residues of the thioredoxin thereby preventing the inhibitor protein Txnip from binding to it during high glucose levels which is to be revealed yet. Thus a clear study over the signaling mechanism behind the anti-oxidative effect of gold nanoparticles that allude to their anti-hyperglycemic role in diabetic conditions would pave way to the quest behind the clinical implication of gold nanoparticles in diabetic treatments and may render it uniquely beneficial as an agent of therapeutic choice for diverse complications.

## Conclusion

Nanotechnology is undergoing explosive expansions in many areas serving mankind, due to which even poorer developing countries have also decided that this new technology could represent a considered investment in future economic and social well-being that they cannot ignore. The gold nanoparticles are known for their tremendous applications in the field of theapeutics and diagonosis. In the present study we have confirmed the anti-oxidative and anti-hyperglycemic activities of gold nanopartcles in streptozotocin induced diabetic mice by balancing or inhibiting the ROS generation at hyperglycemic conditions; scavenging free radicals; thus increasing the anti-oxidant defense enzymes. The gold nanoparticles have been proven for their non-toxic and protective effects over the organs, without inducing any lethal effects in the mice model, thereby accomplishing a sustained control over the disease progression. These potential application of gold nanoparticles in preventing oxidative stress and their adverse effects, induced at hyperglycemic conditions has opened up way for a new resource of cost economic alternative in the treatment of diabetic progression. Furthurmore, a clear study over the mechanism and the downstream pathways through which the gold nanoparticles influenze the control over the anti-oxidant systems and their reverse effect over hyperglycemic conditions may solely contribute to its future therapeutic applications in diabetes mellitus.

## Methods

### Synthesis of Gold nanoparticles

Gold nanoparticles (AuNPs) of 50 nm were synthesized based on the method previously reported with slight modifications [56,57]. In a typical experiment, 2 g of wet *Bacillus licheniformis *biomass was taken in an Erlenmeyer's flask. 1 mM HAuCl_4 _solution was prepared using deionized water and 100 ml of the solution mixture was added to the biomass. Then the conical flask was kept in a shaker at 37°C (200 rpm) for 24 h for the synthesis of nanoparticles.

### Characterization of the AuNPs

Characterization of synthesized and purified nanoparticles was carried out according to the methods described previously [58,59]. The samples to be analyzed for transmission electron microscopy (TEM) analysis were prepared on carbon-coated copper TEM grids. TEM analysis was performed on a JEOL model 1200EX instrument, Japan, operated at an accelerating voltage of 120 kV. The as-synthesized samples were then checked for the structure and phase purity based on the X-ray diffraction (XRD) analysis using a Bruker AXS D8 Advance Powder X-ray diffractometer (using CuKαλ = 1.5418Åradiation).

### Endotoxin assay

The Millipore H_2_O, used in all the experiments in our research, was tested for endotoxins using the Gel clot method according to manufacturer's instructions (Lal endotoxin assay kit). Formation of gel-clot when sample treated according to the kit manufacturer indicated the presence of endotoxin in a sample analyzed. Similarly, prior to treatment in mice, the nanoparticles suspension in deionized water was checked for possible endotoxin contamination.

### Determination of concentration of the gold nanoparticles

The concentration of gold nanoparticles to be administered in nM level was determined by the method which has been previously reported [60]. The calculation is as follows

Initially the average number of atoms per nanoparticles was calculated using the formula

$$N=\frac{\pi \rho D^{3}}{6\;M}N_{A}$$

Where, N = number of atoms per nanoparticles, π = 3.14, ρ = density of face centered, cubic (fcc) gold = 19.3 g/cm^3^, D = average diameter of nanoparticles = 50 nm = 50 × 10^-7 ^cm, M = atomic mass of gold = 197 g, N_A _= number of atoms per mole (Avogadro's Number) = 6.023 × 10^23^

$$\begin{array}{c} \text{N}=\left[\pi \times \text{19}.\text{3}\times {\left(\text{5}0.0\times \text{1}0^{-\text{7}}\right)}^{\text{3}}\times \text{6}.0\text{23}\times \text{1}0^{\text{23}}\right]\\ \text{6}\times \text{197}\\ \text{i}.\text{e}.\text{N}=\text{3862}00\text{27}.\text{74} \end{array}$$

then the molar concentration of the nanoparticles solution was determined by

$$C=\frac{N_{T}}{NVN_{A}}$$

Where, C = molar concentration of nanoparticle solution, N_T _= Total number of gold atoms added as AuCl_4_^- ^= 1 M, N = number of atoms per nanoparticle (from calculation1), V = volume of the reaction solution in L, N_A _= Avogadro's number = 6.023 × 10^23^

$$\begin{array}{l} \text{C}=\frac{\left[1\times 6.023\times 10^{23}\right]}{38620027.74\times 1\times 6.023\times 10^{23}}\\ \text{C}=2.589\times 10^{-8}\text{M}/\text{L}=2589.3\text{ nM}/10\text{ml} \end{array}$$

Further, the required concentration were made out from the obtained values

### Selection of animals

The study was conducted on male albino mice of 5 to 6 weeks age, weighing 35 ± 5 g, housed in polycarbonate cages (five mice per cage) at an ambient temperature of 25 ± 2°C with 12 h-light and 12 h-dark cycle. The mice were fed with commercially obtained rodent chow and water ad libitum. The animals were allowed to acclimatize to the laboratory environment and then they were randomly subjected to the experiment. All the experiments on animals were carried out as per the guidelines of the institutional animal ethics committee (509/01/c/CPCSEA). The entire experimental protocols performed in this manuscript have obtained prior approval from the same committee.

### Optimization of Dosage

The synthesized gold nanoparticles were made to a various concentrations such as 100 nM, 200 nM, 300 nM, 400 nM, 500 nM, 600 nM, 700 nM and 800 nM. 3 mL of each of these solutions were centrifuged at 14,000 rpm for 20 mins separately and the pellet obtained was resuspended in 1.2 mM sodium citrate that resulted in the following concentrations of gold nanoparticles such as 0.5 mg/mL, 1 mg/mL, 1.5 mg/mL, 2 mg/mL, 2.5 mg/mL, 3 mg/mL, 3.5 mg/mL and 4 mg/mL respectively.

These various concentrations of nanoparticles were treated in the mice for a period of 15 days at a dosage of 0.5 mg, 1 mg, 1.5 mg, 2 mg, 2.5 mg, 3 mg, 3.5 mg and 4 mg/kilogram body weight/day and their ability to control the blood glucose level in the diabetic mice by daily assessment of the blood glucose from the tail vein. Based on the assessment made on the final day the Effective inhibitor dosage (EC_50_) 2.5 mg/kg.b.wt/day of gold nanoparticles which reduced the blood glucose level significantly [data not shown] in comparison to other dosages was fixed as the optimum dosage, with which the further studies were carried out.

### Toxicity studies

In this study the mice were randomly divided into two groups (n = 6 mice per group)

Group 1 - Control

Group 2 - AuNPs treated,

Each mouse of Group 2 was administered with gold nanoparticles suspended in deionized water at a dosage of 2.5 mg/kg.b wt/day, through intraperitonial injection with the help of a tuberculin syringe for a period of 15 days. The control group mice were treated with citrate buffer alone. At the end of 15 days treatment all the mice of the two groups were starved over night and were euthanized on the next day to determine the toxicity through examination of hematological and histological analysis.

### Hematological analysis

Blood samples were collected by intra cardiac puncture following anesthesia with ketamine-xylazine. Whole blood was immediately collected in EDTA coated vials for examining hematological toxicity. Hematology analysis includes determination of white blood cell (WBC), Red blood cell (RBC), platelet count, hemoglobin (Hb) level, mean corpuscular hemoglobin (MCH), mean corpuscular hemoglobin concentration (MCHC), mean corpuscular volume (MCV) by the use of automated hematological analyzer (MS9 Differential Cell Counter 3 Part, HD Consortium, India).

### Histopathology

The histological analysis over the toxicity of AuNPs after the treatment for 15 days was performed by examining the morphological changes induced by gold nanoparticles, over the organs such as liver, kidney, lungs and spleen. The organs were collected and fixed with a 10% formalin neutral buffer solution, embedded in paraffin, and cut into 5-μm-thicksections. The fixed sections were stained for analysis using hematoxylin and eosin (H and E) staining. The sections were examined under light microscope and photomicrographs of the fixed organs were obtained.

### Bio distribution of gold nanoparticles

The vital organs (liver, kidney, spleen and lung) were isolated separately from the mice of both the control and gold treated groups. They were then submitted for inductively coupled plasma mass spectrometry (ICP-MS analysis) in order to determine the gold element, and evaluate the bio-distribution of gold nanoparticles in different vital organs. Briefly, the collected organs (liver, kidney, spleen and lungs) were weighed and then dissolved in a 75% HNO_3 _solution (by volume) such that the amount of acid added was equal to ten times the weight of the sample (wet). The samples were then heated overnight at 50°C with occasional venting by loosening the caps. The resulting solution was then diluted with deionized water and the total dilution was made upto about 200 times that of the original weight of the sample. These solutions were again heated overnight at 50°C. Finally, the solutions were cooled, diluted another 10 fold and subsequently analyzed on the Inductively Coupled Plasma Mass Spectrometer (Perkin Elmer: Elan 6100). We used calibration standards at 0.1, 1.0, and 10.0 ppm Au for the analysis. All lines were observed from an axial view point of the plasma to increase sensitivity. Five replicates per sample were used to obtain the standard deviation value. All the samples were re-diluted and analyzed as duplicates to insure the reproducibility.

### Experimental design

The mice were divided into four groups (n = 6 mice per group) as follows

Group I - Normal control mice (nondiabetic, untreated).

Group II - diabetes induced mice used as diabetic control - **DC **(diabetic, untreated).

Group III - Diabetic induced mice treated with AuNPs -**DT**

Group IV - Normal mice treated with AuNPs - **NT**

### Induction of diabetes mellitus and treatment with AuNPs

Diabetes was induced by administering intraperitonial injection of a freshly prepared solution of streptozotocin (STZ) (50 mg/Kg b.wt) in 0.1 M cold citrate buffer (pH 4.5) to the overnight fasted mice of group II and group III. Because of the instability of STZ in aqueous media, the solution was made using cold citrate buffer (pH 4.5) immediately before administration. The mice were allowed to drink 5% glucose solution overnight to overcome the drug-induced hypoglycemia. The blood glucose values were measured to be above 250 mg/dl on the third day after STZ injection thus confirming the induction of diabetes in the mice.

Once the hyperglycemic condition was confirmed the treatment was started on the 5^th ^day after STZ injection and it was considered as 1^st ^day of treatment. The gold nanoparticles were administered to each mouse of group III and group IV at a dosage of 2.5 mg/kg.b.wt/day through intraperitonial injection with the help of a tuberculin syringe for 45 days, while the control group received citrate buffer alone.

### Glucose tolerance test (*GTT*)

The oral GTT was performed after the treatment of gold nanoparticles in group III and IV for 45 days in order to study the control effect of gold nanoparticles over glucose induced tolerance. The mice were fasted over night and blood was collected from the tip of the tail vein for the determination of Fasting Blood Glucose level. OGTT was performed by oral administration of glucose for about 1 g/kg b.wt dissolved in 0.1 ml of clean water to the overnight fasted animals. At various time intervals of 30, 60, 90 and 120 min after the oral glucose load the blood samples were collected from the tail vein with potassium oxalate and sodium fluoride for the estimation of glucose. The results of blood glucose values were expressed in milligrams per deciliter of blood.

### Euthanization of experimental animals

After completion of the FBG and OGTT, the mice of all the experimental groups were deprived of food overnight and euthanized by cervical dislocation under ketamine-xylazine anesthesia. The blood and organ samples were collected carefully for various biochemical estimations.

### Estimation of Blood glucose and Urea

The blood samples collected through cardiac puncture, in sterile vials were immediately used for the estimation of blood glucose level using GOD-POD glucose estimation kit. The values were expressed as mg/dL. The level of urea in the plasma was estimated by the method described earlier [61]. Briefly, to 0.1 ml of plasma, 3.3 ml of water, 0.3 ml of 10% sodium tungstate and 0.3 ml of 0.67 N sulphuric acid reagents were added. The suspensions were centrifuged and to 1 ml of supernatant, 0.4 ml of diacetylmonoxime and 2.6 ml of sulphuric acid-phosphoric acid reagents were added in that order. Standard urea (20-50 μg/ml) was also treated in a similar manner and heated in a boiling water bath for 30 minutes, cooled and the developed colour was measured at 480 nm. The values were expressed as mg/dl of plasma.

### Serum analysis

Whole blood was placed in a clotted vial and centrifuged. The collected serum was submitted for lipid profiling and biochemical analysis to an automated analyzer (Micro Lab 300: Merk, Netherland). Serum biochemical analysis was carried out to determine the level of metabolic enzymes in the liver such as aspartate aminotransferase (AST), alanine aminotransferase (ALT), alkaline phosphates (ALP), acid phosphatase (ACP) in the liver and creatinine stating the normalcy of renal functions.

### Measurement of ROS generation

The ROS generation in the liver was determined using the Nitrotetrazolium blue (NBT) reduction assay as described by method described earlier [62] with minor modifications. The assay is based on the reduction of the yellow water-soluble NBT powder to a blue, insoluble substance upon reduction. Briefly, a known weight of a segment of liver was incubated with 250 μl of NBT solution for 1 h. The reaction was stopped by the addition of one vol. of acetic acid. After centrifugation (1 min at 12000 g), intracellular reduced NBT was solubilized by adding 150 μl of 50% (v/v) acetic acid to the cell pellet followed by vortexing for 5 min. Cell debris was finally pelleted and the absorbance of the supernatant determined at 595 nm in a 96-well plate reader (Biorad, Model 680, Japan).

### Preparation of tissue homogenate

A segment of liver tissue from the four experimental groups (I, II, III, and IV) were excised separately and rinsed in ice cold saline. A known weight of the tissue was homogenized by a tissue homogenizer using a Teflon pestle at 4°C at pH 7.4. The tissue homogenates obtained were centrifuged at 3,000-× g for 10 min at 4°C using Sorvall refrigerated centrifuge. The supernatant was stored in -20°C for further studies.

### SOD, Catalase, GSH and GPx activities in liver

The anti-oxidant system comprise of several enzymes such as catalase, SOD, GSH, GPx etc, responsible for maintaining a balanced system of oxidation reactions thereby inhibiting an up drift in oxidative stress [63].

The SOD activity was measured according to the method described earlier [64]. Briefly, the reaction mixture (2.1 ml) contained 1924 μl sodium carbonate buffer (50 mM), 30 μl Nitrobluetetrazolium (1.6 mM), 6 μl Triton X-100 (10%) and 20 μl hydroxylamine-HCl (100 mM). Subsequently, 100 μl of enzyme source (tissue homogenate) was added and absorbance (560 nm) was read for 5 min. against the blank (reaction mixture without enzyme source). The change in the absorbance was calculated per minute (ie. Δ560) and used in the estimation of enzyme activity.

Reduced glutathione content was measured as described by method earlier [65]. Briefly, the reaction mixture containing 1.2 ml EDTA (0.02 M), 1 ml distilled water, 250 μl 50% trichloroacetic acid and 50 μl Tris buffer (0.4 M, pH 8.9) was centrifuged at 300 xg for 15 min. The clear supernatant (500 μl) was mixed with 1 ml of 0.4 M Tris buffer (containing 0.02 M EDTA, pH 8.9), 100 μl of 0.01 M DTNB [5, 5'-dithio-bis-(-2-nitrobenzoic acid)] and 100 μl enzyme source. The mixture was incubated at 37°C for 25 min. The yellow color developed was read at 412 nm against a blank.

The level of catalase was assayed according to the method described earlier [66]. Briefly 1.2 ml of the phosphate buffer was added to 0.2 ml of tissue homogenate and the enzyme reaction was initiated by the addition of 1.0 ml of H_2_O_2 _solution. The decrease in the absorbance was measured at 420 nm at 30 seconds intervals for minutes. The enzyme blank was run simultaneously with 10 ml of distilled water instead of hydrogen peroxide. The enzyme activity was expressed as μmoles of H_2_O_2 _decomposed/min/mg protein.

Glutathione peroxide (GPx) activity was assayed according to the method described earlier [67]. Briefly, the reaction mixture consisted of 0.2 ml of EDTA, 0.1 ml of sodium azide, 0.1 ml of H_2_O_2, _0.2 ml of reduced glutathione, 0.4 ml of phosphate buffer and 0.2 ml tissue homogenate were incubated at 37°C for 10 minute. The reaction was arrested by addition of 0.5 ml of TCA and the tubes were centrifuged at 5000 rpm and the color developed was read at 420 nm immediately. The enzyme activity was expressed as μmoles of glutathione oxidized/min/mg protein.

### Lipid peroxidation estimation

The method described earlier was used [68] to estimate the lipid peroxidation that took place during hyperglycemia induced oxidative stress leading to ROS generation. Briefly, a reaction mixture was prepared by using, 10 μl ferrous sulfate (100 mM), 10 μl ascorbic acid (150 mM), 100 μl Tris. buffer (150 mM, pH 7.1), 780 μl distilled water and 100 μl enzyme source so that the final volume is 1.0 mL. Then the reaction mixture was incubated at 37°C for 25 min. Thiobarbituric acid (0.375%, 2 ml) was then added to the mixture and allowed to react at 100°C (in water bath) for 15 min. The reaction mixture was then centrifuged (800 × g for 10 min.) and the absorbance values of the supernatant obtained were measured at 532 nm against a blank.

### Histological studies of the experimental animals

The pancreas plays a crucial role in the effective utilization of blood glucose and the liver is known for possession of the major content of the vital enzymes that are responsible for the normal metabolic activities in the system. Any disruptions or degenerations in these two organs may lead to several metabolic disorders mainly diabetes mellitus. Thus the pancreas and the liver of the four experimental groups were carefully dissected out. They were fixed with a 10% formalin neutral buffer solution, embedded in paraffin, and cut into 5-μm-thicksections. The sections obtained were stained using hematoxylin and eosin and they were examined under light microscope. The photomicrographs of them were obtained.

### Statistical analysis

Values were expressed as mean ± S.D. The statistical significance was evaluated by one-way ANOVA followed by Students-'t' test at 5% level of significance between control (p < 0.05) (Graph Pad, San Diego, CA)

## Competing interests

The authors declare that they have no competing interests.

## Disclaimer

The opinions expressed in this article are those of the authors and do not necessarily represent any agency determination or policy.

## Authors' contributions

SB, KK and MS performed the majority of the experiments. SG, SB and KK involved in writing the manuscript. SG and SHE co-ordinated experiments and provided important advice for the experiments along with financial support. SG, HSY, SB, KK and SRKP were involved with the design, interpretation and data analysis. All authors read and approved the final manuscript.

## References

[B1] Aylward GW Progressive changes in diabetics and their management Eye 2005 19 10 1115 1118 10.1038/sj.eye.6701969 16304592

[B2] Wild S Roglic G Green A Sicree R King H Global prevalence of diabetes Diabetes Care 2004 27 1047 1053 10.2337/diacare.27.5.1047 15111519

[B3] Aronson D Hyperglycemia and pathobiology of diabetic complications Adv Cardiol 2008 45 1 16 full_text 18230953 10.1159/000115118

[B4] Harrison D Griendling KK Landmesser U Hornig B Drexler H Role of oxidative stress in atherosclerosis Am J Cardiol 2003 91 3A 7A 11A 10.1016/S0002-9149(02)03144-2 12645638

[B5] Dash D Shrivastava S Applying Nanotechnology to Human Health: Revolution in Biomedical Sciences J Nanotechnology in press doi: ***10.1155/2009/184702***

[B6] Norton S A brief history of potable gold Mol Interv 2008 8 3 120 125 10.1124/mi.8.3.1 18693188

[B7] Jeon KI Byun MS Jue DM Gold compound auranofin inhibits Ikappa B kinase (IKK) by modifying Cys-179 of IKKbeta subunit Exp Mol Med 2003 35 61 66 12754408 10.1038/emm.2003.9

[B8] Kim NH Lee MY Park SJ Choi JS Oh MK Kim IS Auranofin blocks interleukin-6 signalling by inhibiting phosphorylation of JAK1 and STAT3 Immunology 2007 122 607 614 10.1111/j.1365-2567.2007.02679.x 17645497 PMC2266044

[B9] Shah ZA Vohora SB Antioxidant/Restorative Effects of Calcined Gold Preparations Used in Indian Systems of Medicine against Global and Focal Models of Ischaemia Pharmacol Toxicol 2002 90 254 259 10.1034/j.1600-0773.2002.900505.x 12076306

[B10] Kalishwaralal K Deepak V Pandian SRK Gurunathan S Biological synthesis of gold nanocubes using Bacillus licheniformis Bioresour Technol 2009 100 5356 5358 10.1016/j.biortech.2009.05.051 19574037

[B11] Guo R Song Y Wang G Murray RW Does core size matter in the kinetics of ligand exchanges of monolayer-protected Au clusters? J Am Chem Soc 2005 127 2752 2757 10.1021/ja044638c 15725033

[B12] Mukherjee P Bhattacharya R Wang P Wang L Basu S Nagy JA Atala A Mukhopadhyay D Soker S Antiangiogenic properties of gold nanoparticles Clin Cancer Res 2005 11 3530 3534 10.1158/1078-0432.CCR-04-2482 15867256

[B13] Marquis BJ Love SA Braun KL Haynes CL Analytical methods to assess nanoparticle toxicity Analyst 2009 134 425 439 10.1039/b818082b 19238274

[B14] Reeves CL Romero DG Barria MA Olmedo I Clos A Ramanujam VMS Urayama A Vergara L Kogan MJ Soto C Bioaccumulation and toxicity of gold nanoparticles after repeated administration in mice Biochemical and Biophysical Research Communications 2010 393 649 655 10.1016/j.bbrc.2010.02.046 20153731

[B15] Longmire M Choyke PL Kobayashi H Clearance properties of nano-sized particles agents: considerations and caveats Nanomedicine 2008 3 703 717 10.2217/17435889.3.5.703 18817471 PMC3407669

[B16] Zamboni WC Concept and clinical evaluation of carrier-mediated anticancer agents Oncologist 2008 13 248 260 10.1634/theoncologist.2007-0180 18378535

[B17] Ruggiero D Lecomte M Michoud E Lagarde M Wiernsperger N Involvement of cell-cell interactions in the pathogenesis of diabetic retinopathy Diabetes Metab 1997 23 30 42 9059764

[B18] McDonagh PF Hokama JY Microvascular perfusion and transport in the diabetic heart Microcirculation 2000 7 163 181 10901496

[B19] Vinik AI Park TS Stansberry KB Pittenger GL Diabetic neuropathies Diabetologia 2000 43 957 973 10.1007/s001250051477 10990072

[B20] Kowluru RA Kennedy A Therapeutic potential of anti-oxidants and diabetic retinopathy Expert Opin Investig Drugs 2001 10 1665 1676 10.1517/13543784.10.9.1665 11772276

[B21] Chang TI Horal M Jain S Wang F Patel R Loeken MR Oxidant regulation of gene expression and neural tube development: Insights gained from diabetic pregnancy on molecular causes of neural tube defects Diabetologia 2003 46 538 545 10.1007/s00125-003-1171-z 12739027

[B22] Mukherjee P Bhattacharya R Wang P Wang L Basu S Nagy JA Atala A Mukhopadhyay D Soker S l:Antiangiogenic properties of gold nanoparticles Clin Cancer Res 2005 11 3530 3534 10.1158/1078-0432.CCR-04-2482 15867256

[B23] Kalishwaralal K Deepak V Pandian SRK Gurunathan S Biological synthesis of gold nanocubes using Bacillus licheniformis Bioresour Technol 2009 100 5356 5358 10.1016/j.biortech.2009.05.051 19574037

[B24] Sheikpranbabu S Kalishwaralal K Venkatraman D Eom SH Park J Gurunathan S Silver nanoparticles inhibit VEGF-and IL-1beta-induced vascular permeability via Src dependent pathway in porcine retinal endothelial cells J Nanobiotechnology 2009 7 8 10.1186/1477-3155-7-8 19878566 PMC2776000

[B25] Coonoor EE Mwakmuka J Gole A Gold nanoparticles are taken up by human cells but do not cause acute toxicity Small 2005 1 325 327 10.1002/smll.200400093 17193451

[B26] Niidome T Yamagata M Okamoto Y Akiyama Y Takahashi H Kawano T Katayama Y Niidome Y PEG-modified gold nanorods with a stealth character for in vivo applications J Control Release 2006 114 3 343 347 10.1016/j.jconrel.2006.06.017 16876898

[B27] Chen YS Ching Y Liau HI Huang GS Assessment of the In Vivo Toxicity of Gold Nanoparticles Nanoscale Res Lett 2009 4 858 864 10.1007/s11671-009-9334-6 20596373 PMC2894102

[B28] Hainfeld JF Slatkin DN Focella TM Smilowitz HM Gold nanoparticles: a new X-ray contrast agent Br J Radiol 2006 79 248 53 10.1259/bjr/13169882 16498039

[B29] Singha SJ Bhattacharya H Datta AK Dasgupta Anti-glycation activity of gold nanoparticles Nanomedicine: NBM 2009 5 21 29 10.1016/j.nano.2008.06.005 18676206

[B30] Williams SB Cusco JA Roddy MA Johnstone MT Creager MA Impaired nitric oxide-mediated vasodilation in patients with non-insulin-dependent diabetes mellitus J Am Coll Cardiol 1996 27 3 567 74 10.1016/0735-1097(95)00522-6 8606266

[B31] Popper H Cholestasis Annu Rev Med 1968 19 39 56 10.1146/annurev.me.19.020168.000351 4871695

[B32] Hainfeld JF Slatkin DN Smilowitz HM The use of gold nanoparticles to enhance radiotherapy in mice Phys Med Biol 2004 49 309 15 10.1088/0031-9155/49/18/N03 15509078

[B33] King GL Loeken MR Hyperglycemia-induced oxidative stress in diabetic complications. Histochem Cell Biol 2004 122 333 338 10.1007/s00418-004-0678-9 15257460

[B34] Giugliano D Ceriello A Oxidative stress and diabetic vascular complications Diabetes Care 1996 19 257 267 10.2337/diacare.19.3.257 8742574

[B35] Feldman EL Stevens MJ Greene DA Pathogenesis of diabetic neuropathy Clin Neurosci 1997 4 365 370 9358981

[B36] Ruggiero D Lecomte M Michoud E Lagarde M Wiernsperger N Involvement of cell-cell interactions in the pathogenesis of diabetic retinopathy Diabetes Metab 1997 23 30 42 9059764

[B37] Yakimovich NO Ezhevskii AA Guseinov DV Smirnova LA Gracheva TA Klychkov KS Antioxidant properties of gold nanoparticles studied by ESR spectroscopy Russian Chemical Bulletin 2008 57 3 520 523 10.1007/s11172-008-0080-1

[B38] Junpingchen S Patil S James F Mcginnis Rare earth nanoparticles prevent retinal degeneration induced by intracellular peroxides Nat. Nanotechnol 2006 1 2 142 50 10.1038/nnano.2006.91 18654167

[B39] Shukla R Bansal V Chaudhary M Basu A Bhonde RR Sastry M Biocompatibility of Gold Nanoparticles and Their Endocytotic Fate Inside the Cellular Compartment: A Microscopic Overview Langmuir 2005 21 23 10644 10654 10.1021/la0513712 16262332

[B40] Onizawa S Aoshiba K Kajita M Miyamoto Y Nagai A Platinum nanoparticle antioxidants inhibit pulmonary inflammation in mice exposed to cigarette smoke Pulm Pharmacol Ther 2009 22 340 9 10.1016/j.pupt.2008.12.015 19166956

[B41] Wang H Wei W Zhang SY Shen YX Yue L Wang NP Xu SY Melatonin-selenium nanoparticles inhibit oxidative stress and protect against hepatic injury induced by Bacillus Calmette-Guérin/lipopolysaccharide in mice J Pineal Res 2005 39 156 63 10.1111/j.1600-079X.2005.00215.x 16098093

[B42] Ceriello A New Insights on Oxidative Stress and Diabetic Complications May Lead to a "Causal" Antioxidant Therapy Diabetes care 2003 26 1589 1596 10.2337/diacare.26.5.1589 12716823

[B43] Scampicchio M Wang J Blasco AJ Arribas AS Mannino S Escarpa A Nanoparticle-Based Assays of Antioxidant Activity Anal Chem 2006 78 2060 2063 10.1021/ac052007a 16536447

[B44] Svedberg EB Ahner J Shukla N Ehrman SH Schilling K FePt nanoparticle hydrodynamic size and densities from the polyol process as determined by analytical ultracentrifugation Nanotechnology 2005 16 953 956 10.1088/0957-4484/16/6/056

[B45] Moghimi SM Exploiting Bone-Marrow Microvascular Structure for Drug-Delivery and Future Therapies Adv Drug Deliv Rev 1995 17 61 73 10.1016/0169-409X(95)00041-5

[B46] Moghimi SM Hunter AC Capture of stealth nanoparticles by the body's defences Crit Rev Ther Drug Carrier Syst 2001 18 527 550 11789674

[B47] Choi HS Liu W Misra P Tanaka E Zimmer JP Ipe BI Bawendi MG Frangioni JV Renal clearance of quantum dots Nature Biotechnol 2007 25 1165 1170 10.1038/nbt1340 PMC2702539 17891134

[B48] Banerjee T Mitra S Singh AK Sharma RK Maitra A Preparation, characterization and biodistribution of ultrafine chitosan nanoparticles Int J Pharm 2002 243 93 105 10.1016/S0378-5173(02)00267-3 12176298

[B49] Svedberg EB Ahner J Shukla N Ehrman SH Schilling K FePt nanoparticle hydrodynamic size and densities from the polyol process as determined by analytical ultracentrifugation Nanotechnology 2005 16 953 956 10.1088/0957-4484/16/6/056

[B50] King GL Loeken MR Hyperglycemia-induced oxidative stress in diabetic complications. Histochem Cell Biol 2004 122 333 338 10.1007/s00418-004-0678-9 15257460

[B51] Nishikawa T Edelstein D Du XL Yamagishi SI Matsumura T Kaneda Y Yorek MA Beebe D Oates PJ Hammes HP Giardino I Brownlee M Normalizing mitochondrial superoxide production blocks three pathways of hyperglycaemic damage Nature 2000 404 787 790 10.1038/35008121 10783895

[B52] Inoguchi T Li P Umeda F Yu HY Kakimoto M Imamura, M Aoki T Etoh T Hashimoto T Naruse M Sano H Utsumi H Nawata H High Glucose Level and Free Fatty Acid Stimulate Reactive Oxygen Species Production Through Protein Kinase C-Dependent Activation of NAD(P)H Oxidase in Cultured Vascular Cells Diabetes 2000 49 1939 1945 10.2337/diabetes.49.11.1939 11078463

[B53] Evans JL Goldfine ID Maddux BA Grodsky GM Oxidative stress and stress-activated signaling pathways: a unifying hypothesis of type 2 diabetes Endocr Rev 2002 23 5 599 622 10.1210/er.2001-0039 12372842

[B54] Lao F Chen L Li W Ge C Qu Y Sun Q Zhao Y Han D Chen C Fullerene nanoparticles selectively enter oxidation-damaged cerebral microvessel endothelial cells and inhibit JNK-related apoptosis ACS Nano 2009 3 3358 68 10.1021/nn900912n 19839607

[B55] Junn E Han SH Im JY Yang Y Cho EW Um HD Kim DK Lee KW Han PL Rhee SG Choi I Vitamin D_3 _Up-Regulated Protein 1 Mediates Oxidative Stress Via Suppressing the Thioredoxin Function Immunol 2000 164 6287 6295 10.4049/jimmunol.164.12.6287 10843682

[B56] Kalishwaralal K Deepak V Pandian SRK Gurunathan S Biological synthesis of gold nanocubes using Bacillus licheniformis Bioresour Technol 2009 100 5356 5358 10.1016/j.biortech.2009.05.051 19574037

[B57] Kalishwaralal K Gopalram S Vaidyanathan R Deepak V Pandian SRK Gurunathan S Optimization of α-amylase production for the green synthesis of gold nanoparticles Colloid Surf B 2010 77 174 80 10.1016/j.colsurfb.2010.01.018 20189782

[B58] Kalimuthu K SureshBabu R Venkataraman D Bilal Mohd Gurunathan S Biosynthesis of silver nanocrystals by Bacillus licheniformis Colloid Surf B 2008 65 150 153 10.1016/j.colsurfb.2008.02.018 18406112

[B59] Kalishwaralal K Deepak V Ramkumarpandian S Nellaiah H Sangiliyandi G Extracellular biosynthesis of silver nanoparticles by the culture supernatant of Bacillus licheniformis Mater Lett 2008 62 4411 4413 10.1016/j.matlet.2008.06.051

[B60] Marquis BJ Love SA Braun KL Haynes CL Analytical methods to assess nanoparticle toxicity Analyst 2009 134 425 439 10.1039/b818082b 19238274

[B61] Natelson S Lugovoy J K Pincus JB Micro estimation of citric acid; A new colorimetric reaction for Pentabromoacetone J Biol Chem 1948 170 597 745 750 18880771

[B62] Cury-Boaventura MF Curi R Regulation of reactive oxygen species (ROS) production by C18 fatty acids in Jurkat and Raji cells Clin Sci 2005 108 245 25 10.1042/CS20040281 15563273

[B63] Baynes JW Role of oxidative stress in development of complications in diabetics Diabetes 1991 40 405 412 10.2337/diabetes.40.4.405 2010041

[B64] Kono Y Generation of superoxide radical during auto-oxidation of dihydroxylamine and an assay for superoxide dismutase Arch Biochem Biophys 1978 186 189 195 10.1016/0003-9861(78)90479-4 24422

[B65] Seldak J Lindsay RH Estimation of total, protein bound and non-protein sulfhydryl groups in tissue with Ellman's reagent Anal Biochem 1968 25 192 205 10.1016/0003-2697(68)90092-4 4973948

[B66] Takahara S Hamilton HB Neel JV Kobara TY Ogura Y Nishimura ET Hypocatalasemia: a new genetic carrier state J Clin Invest 1960 39 610 619 10.1172/JCI104075 13836629 PMC293346

[B67] Rotruck JT Pope Al Ganther HE Swanson AB Selenium biochemical roles as a component of glutathione peroxidase Science 1973 179 588 590 10.1126/science.179.4073.588 4686466

[B68] Beuge JA Aust SV Microsomal lipid peroxidation. *Methods* Enzymol 1978 52 302 310 full_text 10.1016/s0076-6879(78)52032-6 672633

